# Photoplethysmography-Based Machine Learning Approaches for Atrial Fibrillation Burden: Algorithm Development and Validation

**DOI:** 10.2196/78075

**Published:** 2025-11-10

**Authors:** Hong Wang, Binbin Liu, Hui Zhang, Zheqi Zhang, Zhigeng Jin, Hao Wang, Yu-Tao Guo

**Affiliations:** 1 Department of Pulmonary Vessel and Thrombotic Disease, Sixth Medical Center, Chinese PLA General Hospital, 6 Fucheng Road, Haidian District, Beijing, 100048, China, +86-10-66957703; 2 Graduate School of PLA General Hospital, Beijing, China; 3 Department of Cardiology, Second Medical Centre, National Clinical Research Centre for Geriatric Diseases, Chinese PLA General Hospital, Beijing, China

**Keywords:** atrial fibrillation, photoplethysmography, atrial fibrillation burden, wearable devices, arrhythmia

## Abstract

**Background:**

Atrial fibrillation (AF) burden is associated with cardiovascular events such as stroke and heart failure. Recent advancements in photoplethysmography (PPG) technology have provided new insights into noninvasive and convenient AF burden detection.

**Objective:**

This study aimed to establish an AF burden model based on smartwatch-monitored PPG technology to track the progression of AF.

**Methods:**

This prospective pilot study (January 2024 to January 2025) at the Chinese PLA General Hospital enrolled patients with paroxysmal AF. Participants underwent simultaneous rhythm monitoring using smartwatch PPG and 24-hour Holter electrocardiogram monitoring (the gold standard). Five PPG-derived AF burden metrics were defined: (1) ratio of AF episode duration to total monitoring time (M1), (2) ratio of AF episode frequency to total measurements (M2), (3) AF episode density (M3), (4) AF episode variability (M4), and (5) proportion of rapid ventricular rate in AF episodes (>120 beats per minute; M5). Smartwatch PPG signals were collected once per minute. Sensitivity, specificity, accuracy, precision, and *F*_1_ score were used to evaluate the PPG algorithm’s AF detection capability through comparison with the gold standard (24-hour Holter monitoring). The mean absolute error (MAE) and Spearman rank correlation coefficient (*r_s_*) were used to assess the correlation between the PPG-based AF burden metrics and the gold standard.

**Results:**

A total of 145 participants with paroxysmal AF (n=96, 66.2% male; mean age 63.28, SD 14.23 years) were included. Compared to the gold standard, the PPG-based AF burden model demonstrated a sensitivity of 91.5% (95% CI 87.9%-95.1%), specificity of 97.2% (95% CI 95.9%-98.5%), precision of 92.9% (95% CI 88.6%-97.3%), accuracy of 93.3% (95% CI 88.2%-98.5%), and *F*_1_ score of 90.5% (95% CI 86.3%-94.7%). The AF burden model exhibited strong discriminatory power in the test cohort (area under the curve=89.5%, 95% CI 89.4%‐89.7%). For M1, the MAE for the model of AF episode duration as a proportion of total monitoring time was 0.0400 (*P*=.008), with a correlation coefficient (*r_s_*) of 0.8788 (*P*<.001). For M4, the MAE for the AF episode variability model was 3.9967 (*P*<.001), with a correlation coefficient (*r_s_*) of 0.7876 (*P*<.001). The MAE for the average real variability model was 4.6436 (*P*<.001), with a correlation coefficient (*r*_*s*_) of 0.8127 (*P*<.001). The MAE for the average AF change model was 0.3893 (*P*=.27), with a correlation coefficient (*r_s_*) of 0.7246 (*P*<.001).

**Conclusions:**

The PPG-based AF burden model demonstrated high concordance with the gold standard of 24-hour Holter monitoring in tracking AF episode duration and variability, providing new perspectives for exploring AF progression dynamics.

## Introduction

Atrial fibrillation (AF) is one of the most common arrhythmias, associated with an increased risk of cardiovascular adverse events such as stroke and heart failure, and causes significant health, social, and economic burdens [1]. Numerous studies have found a dose-response relationship between AF burden and cardiovascular event risk, making early assessment of AF burden critical to reduce AF complications [2-4]. However, there is currently no standardized definition for AF burden. It generally refers to the percentage of time spent in AF during total monitoring duration. Other studies have alternatively quantified the number of AF episodes by using episode density to define AF burden to explore the relationship between AF burden and cardiovascular adverse events [2,5].

Currently, devices for monitoring AF burden include cardiovascular implantable electronic devices and noninvasive rhythm recorders (such as 24-hour Holter electrocardiograms [ECGs]), both of which demonstrate high diagnostic accuracy. However, these devices face limitations, including requiring hospital-based monitoring under clinician supervision, high costs, and labor-intensive data interpretation by physicians. Recently, photoplethysmography (PPG) has been developed for AF screening and has shown promising accuracy [6,7]. PPG devices for AF screening are commercially available in various formats, including handheld devices, smartwatches, or wristbands. They use built-in optical sensors to monitor blood volume changes in skin capillary beds and estimate heart rhythm via reflected light wavelengths, offering greater comfort and convenience and providing potential opportunities for out-of-hospital ambulatory AF burden assessment [8]. However, the accuracy of PPG-based AF burden detection algorithms in smartwatches remains uncertain [9].

Our study aimed to evaluate the accuracy of smartwatch integrated PPG algorithms in assessing AF burden.

## Methods

### Study Population

Between January 1, 2024, and January 1, 2025, consecutive patients diagnosed with paroxysmal AF were recruited from the Chinese PLA General Hospital. Inclusion criteria were patients aged ≥18 years who provided written informed consent. Exclusion criteria were inability to use wearable devices, cognitive impairment, or presence of implanted cardiac devices (pacemakers or implantable cardioverter-defibrillators).

### Ethical Considerations

This study complied with the World Medical Association’s Declaration of Helsinki and was approved by the Institutional Review Board of the Chinese People’s Liberation Army General Hospital (HZKY-PJ-2023-23). It was also registered with the Chinese Clinical Trial Registry (ChiCTR2300075516). All participants signed the informed consent form before participating in this study. This study strictly adhered to privacy protection protocols in accordance with the Declaration of Helsinki. No financial compensation was provided to participants.

### Signal Acquisition and Processing

This study involved collecting baseline clinical data and PPG signals. Clinical data included demographics, comorbidities, and medications. PPG signals were obtained as follows: after attaching 24-hour Holter ECG electrodes, participants wore smartwatches (Huawei Watch GT2 Pro; Huawei Technologies Co., Ltd.). Simultaneous recordings of cardiac rhythm from both devices were initiated.

### Development and Optimization of the Primary PPG-Based AF Burden Model

The PPG-based AF burden algorithm was developed using 3698 PPG data segments from the previous Mobile Atrial Fibrillation Application (mAFA) study as the training and validation sets [10]. Each data segment had a duration of 1 minute. The classification of the training data is shown in Table 1. The training data segments were each divided into varying durations (8, 16, 24, 32, 40, and 48 seconds) with random start times determined by a random number generator. During model development, we maintained a balanced representation of AF and non-AF episodes across all duration categories, enforced strict subject-level segregation to prevent any overlap between the training and validation datasets, and carefully preserved comparable AF to non-AF ratios in both sets. The raw PPG signal from the sensor module was processed using a bandpass Butterworth digital filter to eliminate low-frequency baseline drift and high-frequency noise, thereby obtaining clear and effective pulsatile PPG waveforms. To overcome limitations of single-time-AF detection algorithms, which exhibit low motion tolerance due to strict signal quality requirements and are inadequate for AF burden assessment, this study proposes a multiscale fusion AF burden detection algorithm featuring continuous PPG acquisition with minute-by-minute interpretation (defining AF burden as >40% of AF beats per minute), high-quality signal extraction from motion corrupted segments, an adaptive length machine learning model for variable duration PPG signals, and context-aware fusion calibration leveraging AF episode continuity to refine single time predictions, thereby achieving accurate AF burden quantification. In this study, we enrolled 145 patients as a test cohort to validate the detection accuracy of the AF burden algorithm compared to 24-hour Holter monitoring.

**Table 1.** The classification of the training data.

Training data category	Record (1-minute segment)
Atrial fibrillation	1090
Premature contraction	540
Sinus rhythm	2067

### Definition of AF Burden

**Figure 1.**
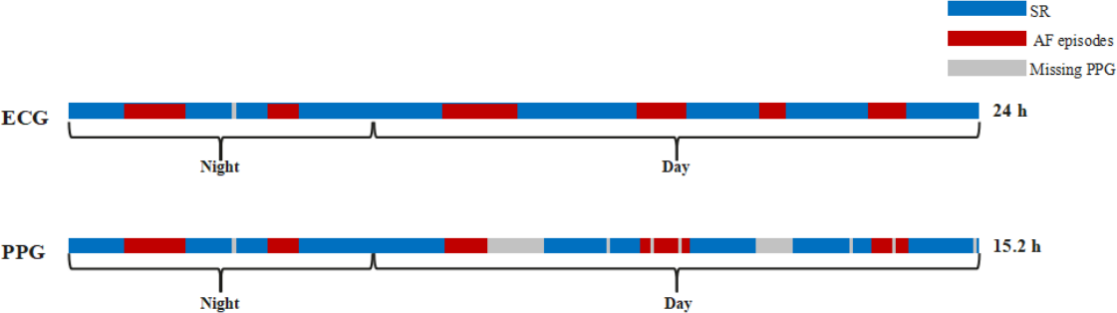
A schematic diagram of metric M1. AF: atrial fibrillation; ECG: electrocardiogram; PPG: photoplethysmography; SR: sinus rhythm.

Metric M1 is the proportion of AF episode duration detected through PPG monitoring relative to the total monitoring time, quantifying temporal AF dynamics (Figure 1).

**Figure 2.**
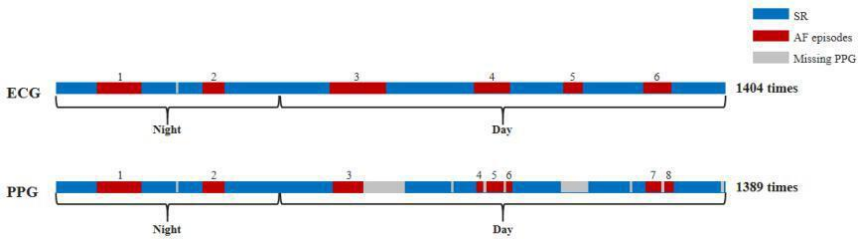
A schematic diagram of metric M2. AF: atrial fibrillation; ECG: electrocardiogram; PPG: photoplethysmography; SR: sinus rhythm.

Metric M2 is the proportion of AF episodes detected through PPG monitoring relative to the total number of monitoring epochs, assessing frequency-based AF variations (Figure 2).

**Figure 3.**
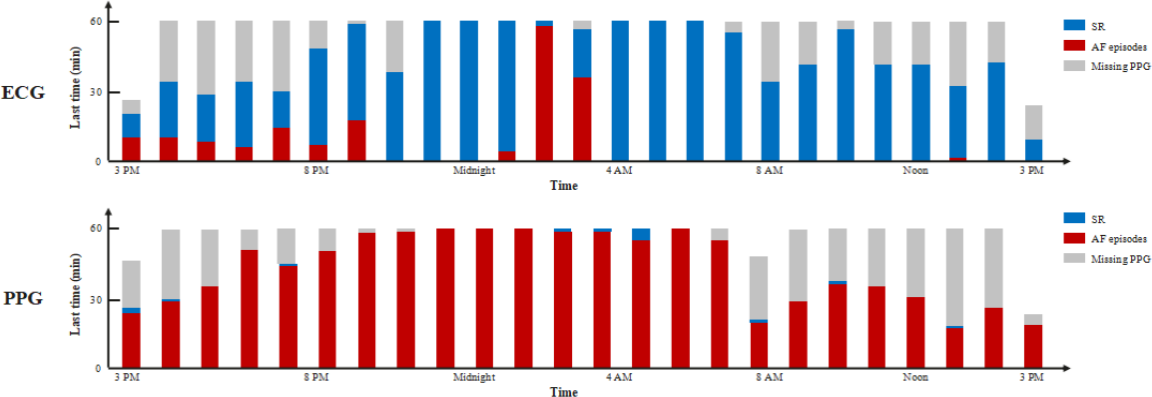
A schematic diagram of metric M3. AF: atrial fibrillation; ECG: electrocardiogram; PPG: photoplethysmography; SR: sinus rhythm.

Metric M3 is the area between the actual AF burden progression curve (derived from PPG monitoring) and the theoretical uniform AF burden development curve, evaluating AF episode clustering (Figure 3).

**Figure 4.**
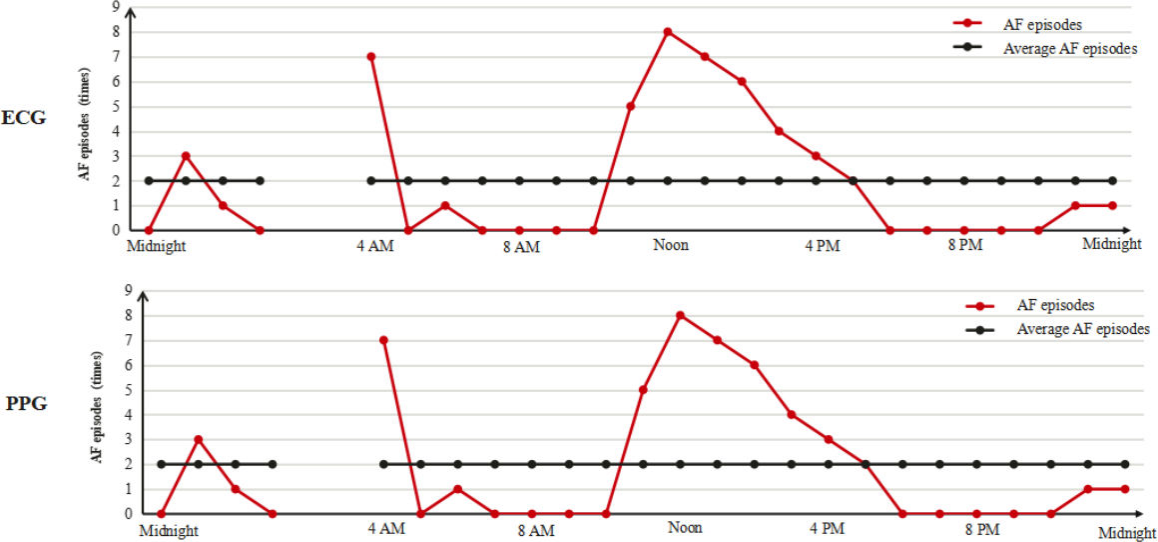
A schematic diagram of metric M4. AF: atrial fibrillation; ECG: electrocardiogram; PPG: photoplethysmography; SR: sinus rhythm.

Metric M4 is a composite metric integrating 3 variability parameters derived from PPG monitoring: AF episode variability (SD of hourly AF counts normalized by 24-hour mean AF frequency), mean real variability (average of SD and mean absolute deviation of hourly AF counts), and mean AF variation (day-night difference in AF counts normalized by overall mean AF frequency). This model quantifies hourly AF burden fluctuations (Figure 4).

**Figure 5.**
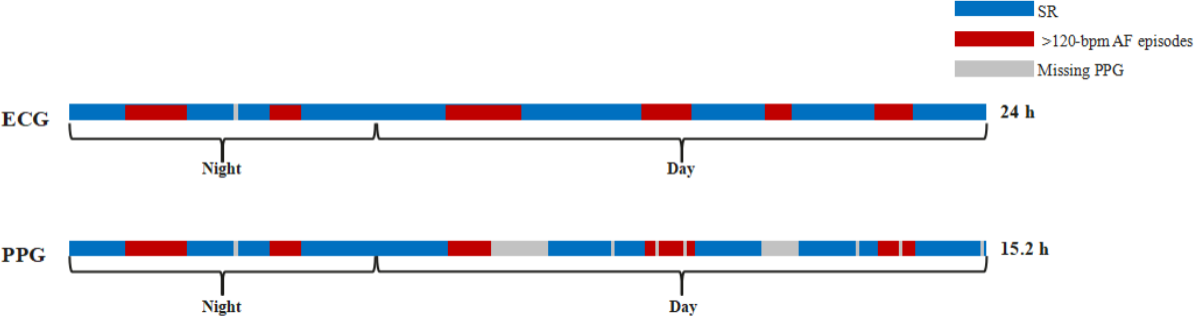
A schematic diagram of metric M5. AF: atrial fibrillation; bpm: beats per minute; ECG: electrocardiogram; PPG: photoplethysmography; SR: sinus rhythm.

**Figure 6.**
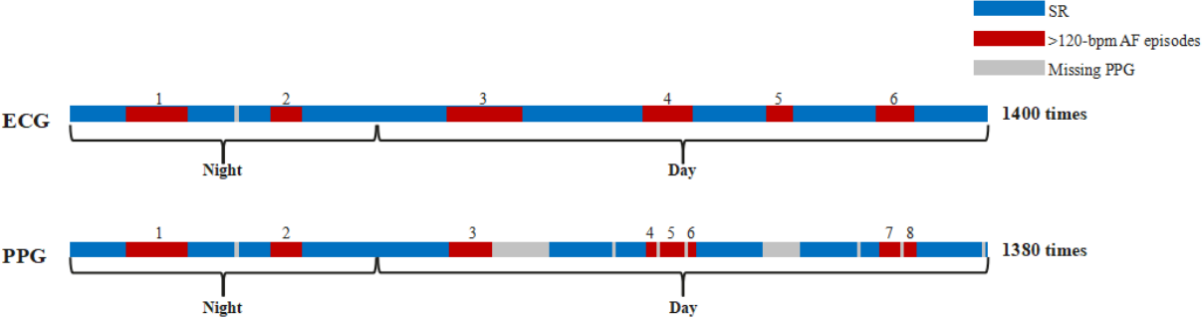
A schematic diagram of metric M5. AF: atrial fibrillation; bpm: beats per minute; ECG: electrocardiogram; PPG: photoplethysmography; SR: sinus rhythm.

Metric M5 quantifies rapid ventricular rate (RVR) AF episodes, defined as the proportion of AF episodes with heart rates of >120 beats per minute (bpm) relative to total monitored AF episodes or the cumulative duration of RVR AF episodes relative to the total monitoring time. This dual parameter model enables characterizing high rate AF burden (Figure 5 and Figure 6).

### Statistical Analysis

Data with a normal distribution were presented as means and SDs. Data with a nonnormal distribution were presented as medians and IQRs and were analyzed using the Mann-Whitney *U* test. Categorical variables were expressed as percentages. Algorithm performance (sensitivity, specificity, accuracy, precision, *F*_1_ score, and area under the receiver operating characteristic curve) was validated against Holter ECG values to assess its capability for continuous AF detection. Correlation between the PPG-based AF burden model and 24-hour Holter ECG monitoring was assessed using the mean absolute error (MAE) and Spearman rank correlation coefficient (*r_s_*; Table 2)

**Table 2.** Clinical significance of each metric.

Performance metric	Formula	Clinical significance
Sensitivity	TP^a^/(TP + FN^b^)	Proportion of actual positive cases correctly identified
Specificity	TN^c^/(TN + FP^d^)	Proportion of actual negative cases correctly excluded
Accuracy	(TP + TN)/(TP + FP + FN + TN)	Proportion of correctly classified cases
Precision	TP/(TP + FP)	Proportion of TPs among all positive test results
*F*_1_-score	2 × TP/(2 × TP + FP + FN)	Balances FNs and FPs when their clinical consequences are comparable
AUC-ROC^e^	—^f^	Overall discriminative ability across all thresholds (0.9=excellent; 0.7‐0.9=moderate)
MAE^g^	$\frac{\sum_{i=1}^{n}\left|p_{i}-g_{i}\right|}{n}$,$p_{i}:PPGg_{i}:ECG$	Quantifies the average prediction error; smaller MAE indicates higher clinical utility for risk stratification or treatment timing
*r_s_* ^h^	—f	Measures monotonic association; high *r*_*s*_ (*r*_*s*_>0.6) suggests that the model captures clinically relevant trends, even if they are nonlinear

a TP: true positive.

b FN: false negative.

c TN: true negative.

d FP: false positive.

e AUC-ROC: area under the receiver operating characteristic curve.

f Not applicable.

g MAE: mean absolute error.

h *r_s_*: Spearman rank correlation coefficient.

A 2-sided *P* value of <.05 was considered statistically significant. The 95% CIs were calculated using the Wilson score method without continuity correction. Analyses were conducted using SPSS Statistics (version 22; IBM Corp) and OpenEpi (version 3.01).

## Results

### Overview

**Figure 7.**
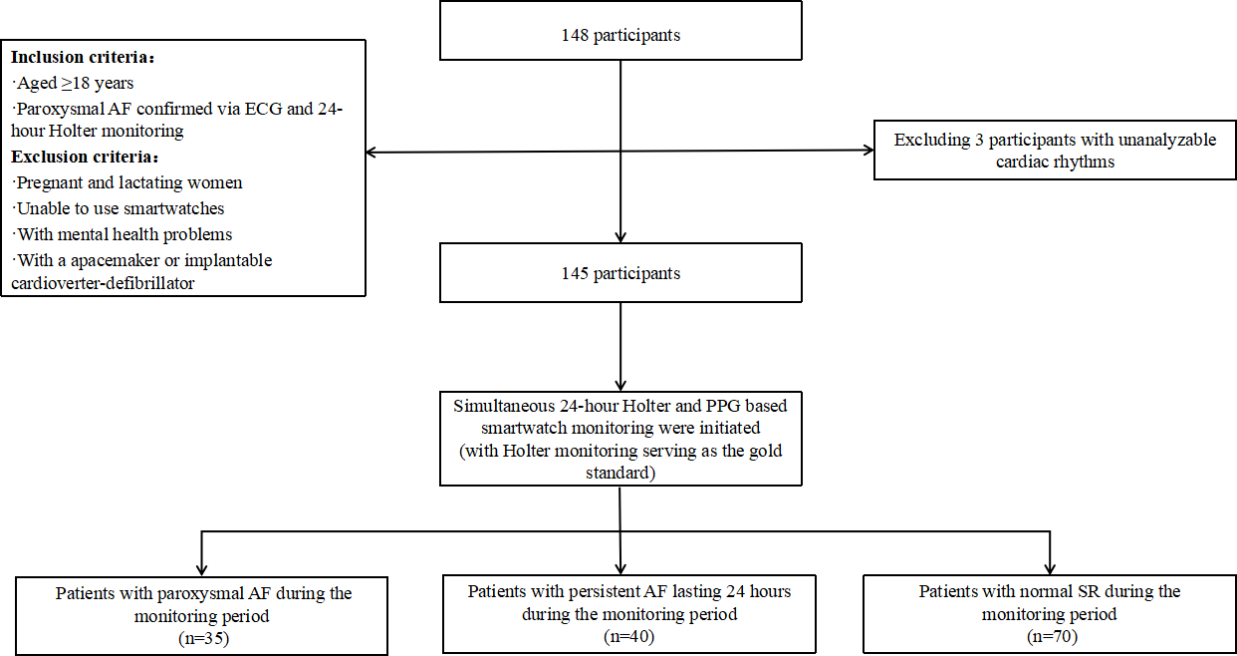
A flowchart of the study. AF: atrial fibrillation; ECG: electrocardiogram; PPG: photoplethysmography; SR: sinus rhythm.

From January 1, 2024, to January 1, 2025, a total of 148 participants were initially enrolled in the study. Of these 148 participants, after excluding 2 (1.4%) with atrial flutter and 1 (0.7%) with atrioventricular block, 145 (98%) were ultimately included in the final analysis (n=96, 66.2% male; mean age 63.28, SD 14.23; range 19 to 90 years). On the basis of expert-reviewed 24-hour Holter ECG monitoring, 75 patients exhibited AF episodes during the monitoring period, including 35 (47%) cases of paroxysmal AF and 40 (53%) cases of persistent AF lasting 24 hours (Figure 7).

### Baseline Characteristics

The study population comprised patients with paroxysmal AF with a mean age of 63.28 (SD 14.23) years, including 66.2% (96/145) male individuals. Baseline characteristics are detailed in Table 3.

**Table 3.** Baseline characteristics of the participants (N=145).

Characteristic	Values
Demographics	
Age (y), mean (SD)	63.28 (14.23)
Male sex, n (%)	96 (66.2)
Medical history, n (%)	
Coronary artery disease	31 (21.4)
Heart failure	4 (2.8)
Hypertension	75 (51.7)
Hyperlipidemia	66 (45.5)
Diabetes mellitus	31 (21.4)
Previous stroke, SE^a^, or TIA^b^	3 (2.1)
Vascular disease	47 (32.4)
Renal dysfunction	7 (4.8)
COPD^c^	9 (6.2)
Hyperthyroidism	3 (2.1)
OSA^d^	15 (10.3)
Anticoagulants, n (%)	
Warfarin	2 (1.4)
Dabigatran	14 (9.7)
Rivaroxaban	21 (14.5)
Apixaban	3 (2.1)
Edoxaban	24 (16.6)
Low–molecular weight heparin	3 (2.1)
Antiarrhythmic drugs, n (%)	
Propafenone	28 (19.3)
Amiodarone	16 (11.0)
Dronedarone	7 (4.8)
Bisoprolol	17 (11.7)
Metoprolol	39 (26.9)

a SE: systemic arterial embolism.

b TIA: transient ischemic attack.

c COPD: chronic obstructive pulmonary disease.

d OSA: obstructive sleep apnea.

**Figure 8.**
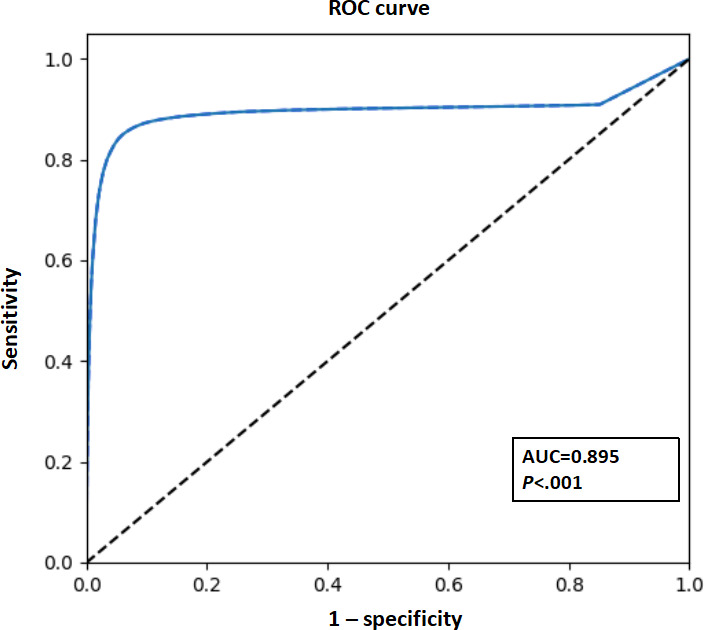
Receiver operating characteristic curves for photoplethysmography–based atrial fibrillation burden model. AUC: area under the curve; ROC: receiver operating characteristic.

The AF burden algorithm demonstrated high performance, with a daily model output rate of 86.6% (95% CI 85.2%-88%). The confusion matrix yielded true positive, false negative, false positive, and true negative counts for PPG signals of 106,975, 11,941, 8787, and 308,082, respectively. Compared to the gold standard, the PPG-based AF burden model demonstrated a sensitivity of 91.5% (95% CI 87.9%-95.1%), specificity of 97.2% (95% CI 95.9%-98.5%), precision of 92.9% (95% CI 88.6%-97.3%), accuracy of 93.3% (95% CI 88.2%-98.5%), and *F*_1_-score of 90.5% (95% CI 86.3%-94.7%). The AF burden model exhibited strong discriminatory power in the test cohort (area under the curve=89.5%, 95% CI 89.4%-89.7%; Table 4). The receiver operating characteristic curve is shown in Figure 8. The performance metrics of the PPG-based AF burden model across varying threshold values are shown in Table 5.

**Figure 9.**
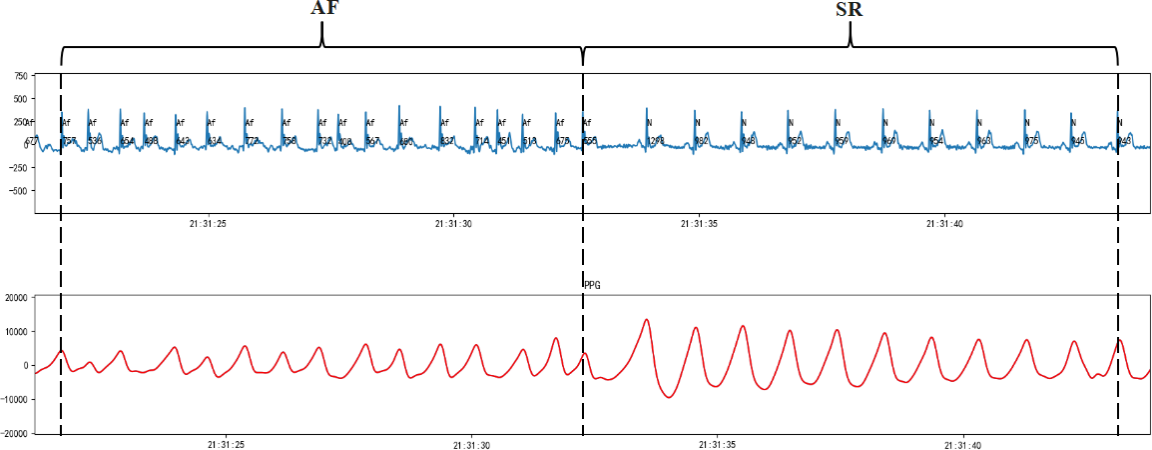
Representative pulse waveform recording from a patient. AF: atrial fibrillation; PPG: photoplethysmography; SR: sinus rhythm.

Figure 9 shows the strong concordance between the PPG and Holter ECG waveforms.

**Table 4.** The performance metrics of the photoplethysmography-based atrial fibrillation burden model.

Performance metric	Performance (%; 95% CI)
Sensitivity	90.0 (87.9‐95.1)
Specificity	97.2 (95.9‐98.5)
Precision	92.9 (88.6‐97.3)
Accuracy	93.3 (88.2‐98.5)
*F*_1_-score	90.5 (86.3‐94.7)
AUC^a^	89.5 (89.4‐89.7)

a AUC: area under the receiver operating characteristic curve.

**Table 5.** The performance metrics of the photoplethysmography-based atrial fibrillation burden model across varying threshold values.

Cutoff point for AF burden threshold	Accuracy	Precision	Sensitivity	Specificity	*F*_1_ score
40%	0.91	0.85	0.84	0.94	0.84
45%	0.92	0.85	0.84	0.94	0.85
50%	0.92	0.84	0.85	0.94	0.85
55%	0.92	0.84	0.86	0.94	0.85
60%	0.92	0.83	0.87	0.94	0.85
65%	0.92	0.83	0.87	0.94	0.85
70%	0.92	0.82	0.88	0.93	0.85

### Evaluation of AF Burden Quantification Performance by the AF Burden Model

#### Metric M1 Evaluation

**Figure 10.**
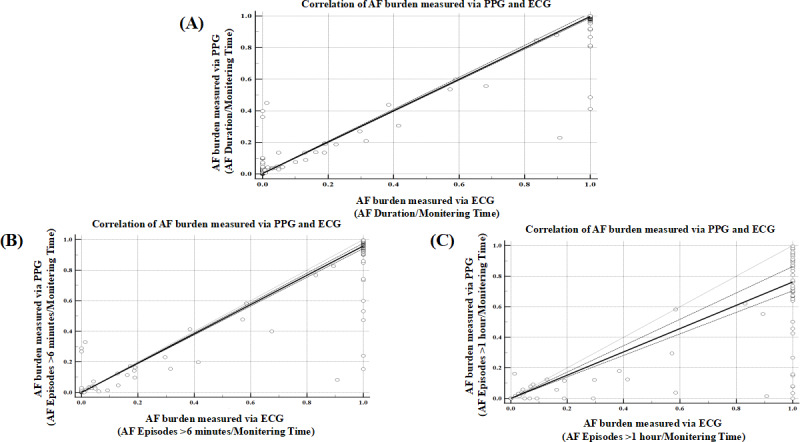
Scatter plot and regression line of AF burden estimated by PPG compared to AF burden estimated by ECG. Each dot represents the AF burden by each method for each patient with AF. Dashed line is the regression line. Solid gray line is line of equality. AF: atrial fibrillation; ECG: electrocardiogram; PPG: photoplethysmography.

For the ratio of AF duration to the total monitoring time, the PPG median was 0.0402 (IQR 0.0086‐0.8671) versus 0.0020 (IQR 0.0086‐0.8671) for 24-hour Holter monitoring, with model performance metrics of MAE=0.0400 (*P*=.008) and *r_s_*=0.8788 (*P*<.001; Figure 10A). For the ratio of AF episodes of >6 minutes to the total monitoring time, the PPG median was 0.0093 (IQR 0.0000‐0.7330) versus 0.0000 (IQR 0.0000‐1.0000) for 24-hour Holter monitoring, with model performance metrics of MAE=0.0582 (*P*=.44) and *r_s_*=0.9233 (*P*<.001; Figure 10B). Regarding the ratio of AF episodes of >1 hour to the total monitoring time, the PPG median was 0.0000 (IQR 0.0000‐0.2947) versus 0.0000 (IQR 0.0000‐1.0000) for 24-hour Holter monitoring, with model performance of MAE=0.1204 (*P*=.04) and *r_s_*=0.9293 (*P*<.001; Figure 10C).

#### Metric M2 Evaluation

**Figure 11.**
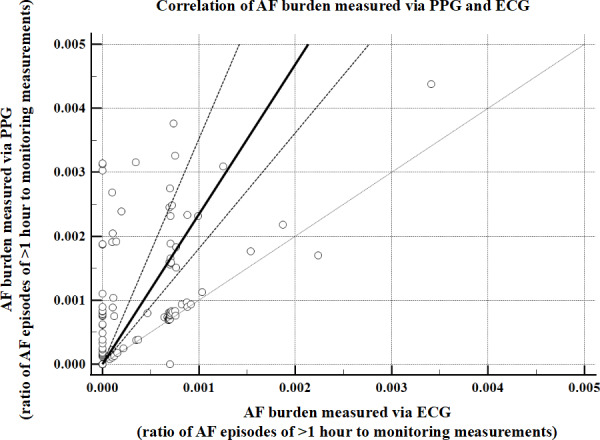
Scatter plot and regression line of AF burden estimated by PPG compared to AF burden estimated by ECG. Each dot represents the AF burden by each method for each patient with AF. Dashed line is the regression line. Solid gray line is line of equality. AF: atrial fibrillation; ECG: electrocardiogram; PPG: photoplethysmography.

The ratio of AF episodes to the total measurements showed a PPG median of 0.0033 (IQR 0.0009‐0.0056) versus 0.0001 (IQR 0.0000‐0.0008) for 24-hour Holter monitoring, with model performance metrics of MAE=0.0320 (*P*<.001) and *r_s_*=–0.0807 (*P*=.33) compared to the gold standard. For the ratio of AF episodes of >6 minutes to the total measurements, the PPG median was 0.0010 (IQR 0.0007‐0.0028) versus 0.0000 (IQR 0.0000‐0.0007) for 24-hour Holter monitoring, with model performance metrics of MAE=0.0015 (*P*<.001) and *r_s_*=0.2360 (*P*=.004). Regarding the ratio of AF episodes of >1 hour to the total measurements, the PPG median was 0.0004 (IQR 0.0000‐0.0009) versus 0.0000 (IQR 0.0000‐0.0007) for 24-hour Holter monitoring, with model performance of MAE=0.0005 (*P*<.001) and *r_s_*=0.7092 (*P*<.001; Figure 11).

#### Metric M3 Evaluation

**Figure 12.**
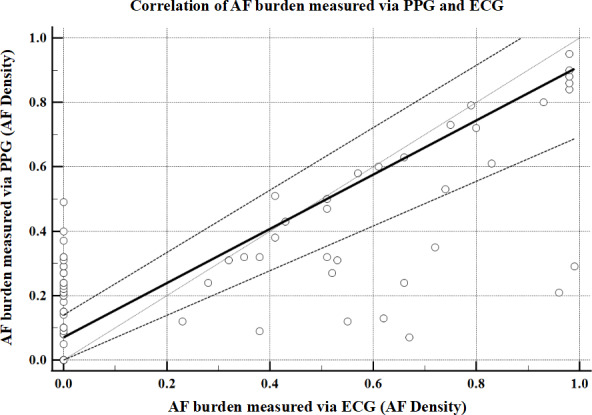
Scatter plot and regression line of AF burden estimated by PPG compared to AF burden estimated by ECG. Each dot represents the AF burden by each method for each patient with AF. Dashed line is the regression line. Solid gray line is line of equality. AF: atrial fibrillation; ECG: electrocardiogram; PPG: photoplethysmography.

The PPG-derived AF density showed a median of 0.2700 (IQR 0.1700-0.4725) compared to 0.0000 (IQR 0.0000-0.5900) for 24-hour Holter monitoring. When validated against the gold standard, the model demonstrated an MAE of 0.1725 (*P<*.001) with an *r_s_* of 0.6576 (*P*<.001; Figure 12)

#### Metric M4 Evaluation

**Figure 13.**
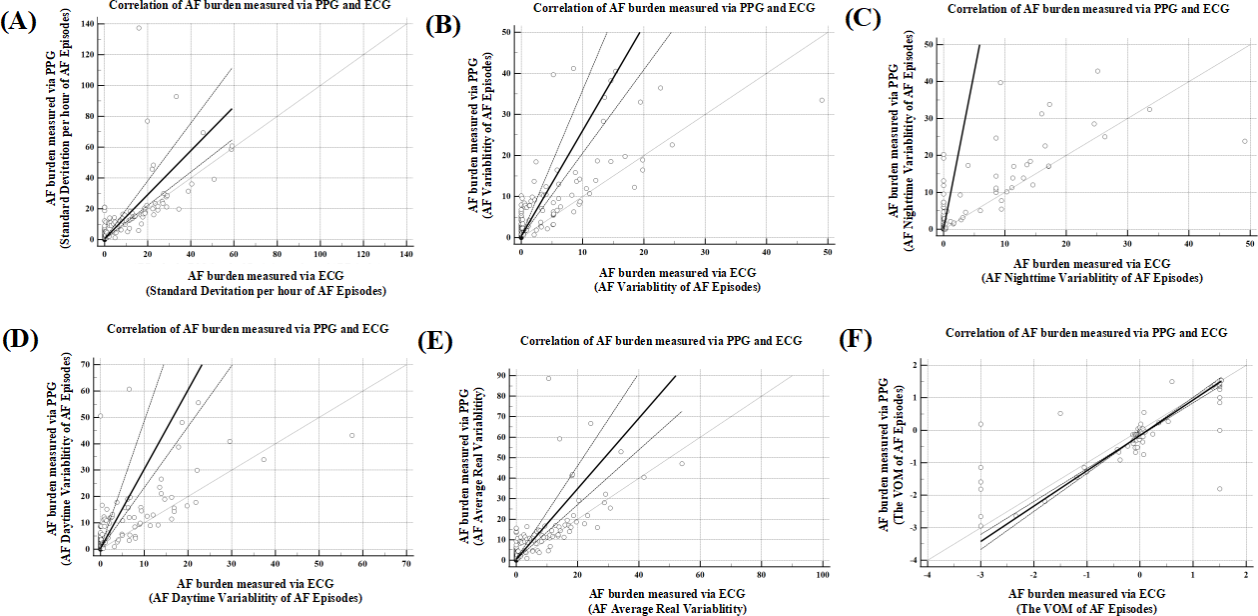
Scatter plot and regression line of AF burden estimated by PPG compared to AF burden estimated by ECG. Each dot represents the AF burden by each method for each patient with AF. Dashed line is the regression line. Solid gray line is line of equality. AF: atrial fibrillation; ECG: electrocardiogram; PPG: photoplethysmography. VOM: variation of the mean (VOM=[∣average AF episode at day−average AF episodes at night∣/average 24-h AF episodes] ×100%).

The SD of hourly AF episodes measured through PPG showed a median of 5.3229 (IQR 1.0375-15.9058) versus 0.4390 (IQR 0.0000-12.8558) for Holter monitoring, with MAE=5.6009 (*P*<.001) and *r_s_*=0.8135 (*P*<.001; Figure 13A). AF variability assessment revealed a median PPG value of 3.3478 (IQR 0.6957-9.2174) compared to 0.1739 (IQR 0.0000-5.2174) for 24-hour Holter monitoring, with MAE=3.9967 (*P*<.001) and *r_s_*=0.7876 (*P*<.001; Figure 13B). Daytime (6 AM-10 PM) variability showed a median PPG value of 3.6000 (IQR 0.8667-11.4667) versus 0.0000 (IQR 0.0000-5.600) for 24-hour Holter monitoring, with MAE=4.8425 (*P*<.001) and *r_s_*=0.7659 (*P*<.001; Figure 13C), whereas nighttime (10 PM-6 AM) variability showed a median PPG value of 1.000 (IQR 0.0000-6.0000) versus 0.0000 (IQR 0.0000-0.2857) for 24-hour Holter monitoring, with MAE=2.6171 (*P*<.001) and *r_s_*=0.6712 (*P*<.001; Figure 13D). Mean real variability measurements yielded a median value of 4.5282 (IQR 0.9031-12.4119) for PPG versus 0.3499 (IQR 0.0000-8.9325) for 24-hour Holter monitoring, with MAE=4.6436 (*P*<.001) and *r_s_*=0.8127 (*P*<.001; Figure 13E). Mean AF variation assessment yielded a median PPG value of 0.0466 (IQR –0.2987 to 1.3361) versus –0.0142 (IQR –0.1249 to 0.0654) for 24-hour Holter monitoring, with MAE=0.3893 (*P*=.27) and *r_s_*=0.7246 (*P*<.001; Figure 13F).

#### Metric M5 Evaluation

For the proportion of AF episodes with a ventricular rate of >120 bpm to total monitored episodes, PPG measurements showed a median of 0.0007 (IQR 0.0007-0.0027) compared to 0.0000 (IQR 0.0000-0.0070) for 24-hour Holter monitoring, with model performance metrics of MAE=0.0151 (*P*=.76) and *r_s_*=0.3435 (*P*<.001). Similarly, for the proportion of time in AF with a ventricular rate of >120 bpm to total monitoring time, the median PPG value was 0.0007 (IQR 0.0007-0.0027) versus 0.0000 (IQR 0.0000-0.0070) for 24-hour Holter monitoring, yielding an identical model performance (MAE=0.0151 and *P*=.76; *r_s_*=0.3435 and *P*<.001). Table 6 summarizes the quantification performance of the AF burden model.

**Table 6.** Photoplethysmography (PPG)–monitored atrial fibrillation (AF) progression features compared to 24-hour electrocardiogram (ECG) monitoring (N=145).

	PPG, median (IQR)	ECG, median (IQR)	MAE^a^	*P* value	*r*_*s*_ ^b^	*P* value
Duration feature						
Ratio of AF duration to total monitoring time (%)	0.0402 (0.0086 to 0.8671)	0.0020 (0.0086 to 0.8671)	0.0400	.008	0.8788	<.001
Ratio of AF episodes lasting over 6 min to total monitoring time (%)	0.0093 (0.0000 to 0.7330)	0.0000 (0.0000 to 1.0000)	0.0582	.44	0.9223	<.001
Ratio of AF episodes lasting over 1 h to total monitoring time (%)	0.0000 (0.0000 to 0.2947)	0.0000 (0.0000 to 1.0000)	0.1204	.04	0.9293	<.001
Number feature						
Ratio of AF episodes to total measurements (%)	0.0033 (0.0009 to 0.0056)	0.0001 (0.0000 to 0.0008)	0.0032	<.001	–0.0807	.33
Ratio of AF episodes lasting over 6 min to total measurements (%)	0.0010 (0.0007 to 0.0028)	0.0000 (0.0000 to 0.0007)	0.0015	<.001	0.2360	.004
Ratio of AF episodes lasting over 1 h to total measurements (%)	0.0004 (0.0000 to 0.0009)	0.0000 (0.0000 to 0.0007)	0.0005	<.001	0.7092	<.001
Aggregation feature						
AF density	0.2700 (0.1700 to 0.4725)	0.0000 (0.0000 to 0.5900)	0.1725	.13	0.6576	<.001
Circadian rhythm feature						
SD per h of AF episodes	5.3229 (1.0375 to 15.9058)	0.4390 (0.0000 to 12.8558)	5.6009	<.001	0.8135	<.001
Variability of AF episodes	3.3478 (0.6957 to 9.2174)	0.1739 (0.0000 to 5.2174)	3.9967	<.001	0.7876	<.001
Daytime AF variability	3.6000 (0.8667 to 11.4667)	0.0000 (0.0000 to 5.6000)	4.8425	<.001	0.7659	<.001
Nighttime AF variability	1.0000 (0.0000 to 6.0000)	0.0000 (0.0000 to 0.2857)	2.6171	<.001	0.6712	<.001
Average real variability^c^	4.5282 (0.9031 to 12.4119)	0.3499 (0.0000 to 8.9325)	4.6436	<.001	0.8127	<.001
VOM^d^ of AF episodes (%)	0.0466 (–0.2987 to 1.3361)	–0.0142 (–0.1249 to 0.0654)	0.3893	.27	0.7246	<.001
Heart rate feature						
Ratio of duration of pulse rate of >120 bpm^e^ to monitoring time (min)	0.0007 (0.0000 to 0.0027)	0.0000 (0.0000 to 0.0070)	0.0151	.76	0.3435	<.001
Ratio of number of pulse rates of >120 bpm to total measurements	0.0007 (0.0000 to 0.0027)	0.0000 (0.0000 to 0.0070)	0.0151	.76	0.3435	<.001

a MAE: mean absolute error; $mae=\frac{\sum_{i=1}^{n}\left|p_{i}-g_{i}\right|}{n}$$p_{i}:PPGg_{i}:ECG$.

b Spearman rank correlation coefficient.

c Average real variability: average AF episodes, SD, and mean absolute deviation per hour were calculated over 24 hours; average real variability was then equal to the average SD and mean absolute deviation over 24 hours.

d VOM: variation of the mean ([∣average AF episodes during the day − average AF episodes at night∣/average 24-hour AF episodes] × 100%; daytime AF variability: 6 AM-10 PM; nighttime AF variability: 10 PM-6 AM).

e bpm: beats per minute.

## Discussion

### Principal Findings

This study confirmed that the PPG-based AF burden model demonstrated strong agreement in tracking AF burden variations, with metric M1 showing optimal performance: for the ratio of AF duration to the total monitoring time, it achieved an MAE of 0.0400 (*P*=.008) and *r_s_* of 0.8788 (*P*<.001); for the ratio of ≥6-minute AF episodes to the total monitoring time, it achieved an MAE of 0.0582 (*P*=.44) and *r_s_* of 0.9233 (*P*<.001); and for the ratio of ≥1-hour AF episodes to the total monitoring time, it achieved an MAE of 0.1204 (*P*=.04) and *r_s_* of 0.9293 (*P*<.001).

The current clinical classification of AF largely relies on symptomatic presentation and ECG findings, categorizing AF into paroxysmal, persistent, long-standing persistent, and permanent types. The management of AF involves CHA_2_DS_2_-VASc (congestive heart failure; hypertension; age of ≥75 years; diabetes mellitus; previous stroke, transient ischemic attack, or thromboembolism; vascular disease; age of 65-74 years; and sex category)–guided thrombotic risk stratification for anticoagulation decisions. However, current classifications inadequately capture AF progression dynamics [9,11]. For instance, in real-world settings, it remains unclear whether frequent, short-duration AF episodes confer a higher thrombotic risk than infrequent but prolonged episodes. Currently, no universally accepted AF burden definition exists, although it is commonly quantified as the percentage of time in AF during monitoring [12]. Alternative definitions incorporate AF episode frequency, density, and temporal variability [2,5,13], reflecting methodological heterogeneity. While implantable loop recorders and 24-hour Holter monitoring are widely used for AF burden assessment, implantable loop recorders are invasive, costly, and may overestimate AF burden by misclassifying atrial arrhythmias as AF [14]. Conversely, 24-hour Holter monitoring underestimates burden due to short duration and tolerability limitations, restricting its utility in daily practice [15]. This study leveraged PPG integrated smartwatches to enable multidimensional ambulatory AF monitoring, innovatively characterizing AF burden across 5 distinct dimensions: temporal duration, episode frequency, density, variability, and RVR. This approach offers novel insights for establishing a PPG-based definition of AF burden.

In recent years, wearable PPG devices have gained traction for AF screening owing to affordability and usability [8,16]. Previous studies have validated PPG’s diagnostic accuracy in AF detection [17-20]. Our team’s previous research (N=187,912) confirmed the feasibility and utility of PPG-based wristbands and smartwatches in population level AF screening, facilitating early AF identification and intervention [7]. In a large cohort (N=1,187,381; mean follow-up 255 days), 93.6% of PPG suspected AF cases were verified, demonstrating the reliability and accuracy of this approach [21]. Despite PPG’s utility in AF screening, its burden monitoring potential is underexplored. Väliaho et al [8] evaluated PPG-based algorithms for continuous AF burden assessment in 173 participants, finding 30-minute intervals as optimal (*F*_1_ score=0.95; sensitivity=94.9%; specificity=98.6%) when comparing 10-, 20-, 30-, and 60-minute reporting intervals. Avram et al [22] evaluated a Samsung smartwatch algorithm (5-minute intervals: sensitivity=87.8%, 95% CI 83.6%-91%; specificity=97.4%, 95% CI 97.1%-97.7%; area under the curve=93.3%), showing strong AF burden correlation (*R*^2^=0.986). These findings suggest PPG’s potential for AF burden quantification, although detection precision for brief AF episodes requires refinement. In this study, we developed a PPG-based AF burden algorithm integrated into smartwatch devices. When evaluated via 1-minute analysis epochs against standard 24-hour Holter monitoring as the reference, the algorithm demonstrated a sensitivity of 91.5% (95% CI 87.9%-95.1%), specificity of 97.2% (95% CI 95.9%-98.5%), and overall accuracy of 95.7% (95% CI 94%-97.4%). Previous studies have reported PPG signal limitations from motion artifacts and other interfering factors, leading to substantial data exclusion. Reissenberger et al [23] reported that approximately 50.7% of PPG signals were classified as noise, necessitating exclusion. To address these challenges, we implemented algorithm optimizations through a novel multiscale fusion approach for AF burden detection, achieving a daily model output rate of 0.86 (95% CI 0.852-0.880) and markedly improving data usability. In this study, metric M1 demonstrated optimal performance in tracking AF duration, consistent with previous findings, showing an MAE of 0.0400 (*P*=.008) and *r*_*s*_ of 0.8788 (*P*<.001) for the ratio of AF duration to total monitoring time and an MAE of 0.1204 (*P*=.04) and *r_s_*=0.9293 (*P*<.001) for the ratio of episodes of >1 hour to total monitoring time. While current studies typically use invasive devices or 24-hour Holter monitoring to define AF burden through episode frequency and density, our study pioneered PPG-based AF burden models integrated into smartwatches. Compared with standard 24-hour Holter monitoring, the AF burden model exhibited higher MAE values, along with a weaker *r*_*s*_, in assessing metrics M2 and M5. This may be attributed to PPG signal interference caused by poor pulse waveform quality, motion artifacts, and noise, which could reduce the models’ accuracy in evaluating AF episode frequency. However, metric M3 demonstrated good correlation and a weaker MAE, with an MAE of 0.1725 (*P*=.13) and *r_s_*=0.6576 (*P*<.001), although further optimization is still needed in the future. Notably, metric M4 demonstrated excellent agreement with the gold standard in tracking AF variability, providing novel insights for assessing temporal AF fluctuations.

AF’s rising prevalence and mortality exacerbate global health burdens, with its association to adverse cardiovascular events including stroke, heart failure, and death potentially linked to AF burden [1,24]. The Catheter Ablation Versus Standard Conventional Treatment in Patients With Left Ventricular Dysfunction and Atrial Fibrillation trial demonstrated that an AF burden threshold of 50% was associated with significant functional and structural changes in cardiomyocytes [25]. There is accumulating evidence suggesting that AF burden is correlated with adverse cardiovascular events, including stroke and heart failure [2,4,26]. A large-scale study (N=39,710) defined AF burden as the ratio of daily AF duration percentage to total monitoring time [13] and the longest single AF episode duration, revealing dose-dependent associations between increasing baseline AF burden and adverse outcomes at the 1- and 3-year follow-ups. A high AF burden may be a significant risk factor for mortality. However, the causal role of AF burden in stroke events and the optimal threshold (ranging from 1 minute to 24 hours) remain unclear. Moreover, the relationship between AF episode frequency, AF density, AF episode variability, and the proportion of RVR AF and adverse cardiovascular events remains unexplored. However, in this study, metrics M1 and M4 demonstrated strong correlation compared to 24-hour Holter monitoring. This provides a novel methodological foundation for future research into the association between PPG-based AF burden detection and adverse cardiovascular events.

While there is evidence suggesting that anticoagulation therapy may be considered based on atrial high rate episode burden [27], the optimal treatment strategy according to specific AF burden thresholds requires further investigation. Our study’s metric M1 for temporal AF burden monitoring showed strong correlation with 24-hour Holter monitoring, potentially offering a novel methodology for future large-scale clinical trials investigating anticoagulant guidance by AF burden quantification. Current AF guidelines recommend active rhythm control to alleviate symptoms in patients with AF, with antiarrhythmic drug therapy and more effective catheter ablation techniques demonstrating the capability to prevent AF recurrence, improve symptoms, and reduce AF burden [28-30]. Drexler et al [31] retrospectively demonstrated that early postprocedural low root mean square of successive differences values following pulmonary vein isolation independently predicted AF recurrence (hazard ratio=0.50; *P*<.001), whereas Zhu et al [32] prospectively identified postablation root mean square of successive differences and percentage of successive normal sinus intervals that differ by more than 50 milliseconds as independent predictors of recurrence in 102 patients with paroxysmal AF. Collectively, recent randomized trials support the beneficial impact of dynamic AF burden monitoring and reduction on clinical outcomes [33]. Our study’s metric M4 exhibited strong correlation with 24-hour Holter monitoring in assessing AF variability, providing a potential technical foundation for future rhythm management strategies guided by AF burden quantification and prediction of postablation recurrence risk.

There is emerging evidence suggesting that AF burden may contribute to adverse cardiovascular outcomes, including stroke and heart failure, necessitating refined assessment methods for optimized risk prediction. While real-world evaluation of AF burden remains uncertain, incorporating this parameter into clinical decision making could enable more precise risk stratification and therapeutic selection. Our study developed a novel smartwatch-based PPG algorithm showing strong concordance with 24-hour Holter monitoring in long-term AF burden assessment. This innovative model incorporates 5 dimensions—temporal AF duration, episode density, frequency, variability, and proportion with rapid ventricular response—enabling multidimensional AF evaluation. This approach provides new insights for investigating PPG-derived AF burden’s relationship with cardiovascular outcomes, potentially informing early intervention strategies, anticoagulation decisions, and rhythm management protocols.

### Limitations

This study has several limitations that should be acknowledged. First, the relatively modest sample size warrants validation in larger, real-world cohorts to ensure generalizability. In addition, PPG technology remains significantly susceptible to motion artifacts and noise interference, particularly during daily activities and hand movements, which may compromise accurate quantification of AF episodes. This technical limitation presents a notable challenge in assessing RVR AF. However, ongoing advancements in wearable device technology and detection algorithms are expected to mitigate these limitations. Second, while current PPG applications are limited to AF screening and cannot provide definitive diagnosis, our preliminary research demonstrated the diagnostic feasibility of integrated single-lead electrocardiograph technology. Future investigations should prioritize large-scale, multicenter randomized controlled trials to systematically evaluate the clinical utility of combining PPG with iECG technology for comprehensive AF burden monitoring.

### Conclusions

A PPG-based AF burden model incorporating dimensions such as duration, frequency, density, variability, and RVR demonstrated good consistency compared to 24-hour Holter monitoring, with optimal tracking performance observed in the duration and variability dimensions. This approach provides technical support for the at-home multidimensional quantification of AF occurrence and progression, offering new insights for defining PPG-based AF burden. However, further optimization and validation are required for the real-world application of this PPG-based AF burden model.

## Abbreviations

AF: atrial fibrillation
bpm: beats per minute
ECG: electrocardiogram
MAE: mean absolute error
mAFA: Mobile Atrial Fibrillation Application
pNN50: percentage of successive normal sinus intervals that differ by more than 50 milliseconds
PPG: photoplethysmography
RVR: rapid ventricular rate
